# Gene delivery to the mouse brain: Facilitating disease modeling and gene therapy

**DOI:** 10.4103/NRR.NRR-D-25-01793

**Published:** 2026-01-27

**Authors:** Osama Ahmed, Aliaa Elshamy, Abraam Yakoub

**Affiliations:** Department of Medicine, Brigham and Women’s Hospital, Boston, MA, USA; Department of Biomedical Engineering, University of North Dakota, Grand Forks, ND, USA; Department of Microbiology and Immunology, Faculty of Pharmacy, Heliopolis University, Cairo, Egypt

Gene delivery to the mouse brain is essential for neural circuit probing or therapeutic testing. We describe viral approaches for gene delivery to the mouse brain, and outline the applications and limitations of each approach, and strategies to mitigate the limitations. We provide a decision-oriented guide to the optimal vector and route combinations for the mouse brain, to facilitate studying brain functions or testing gene therapies *in vivo*.

Gene delivery may allow controlled gene expression in specific neural populations, enabling insights into neural circuit function, neurodevelopment or neurodegeneration mechanisms (Luo et al., 2018). Gene delivery also represents a potential therapeutic approach for diseases, such as delivering therapeutic constructs or CRISPR gene-editing reagents (gRNA and Cas), and has the potential to treat conditions ranging from monogenic disorders such as spinal muscular atrophy, to complex neurodegenerative disorders such as Alzheimer’s disease and Parkinson’s disease. Current methodologies to central nervous system (CNS) gene delivery leverage advanced viral vector systems with engineered tropism, innovative delivery routes, and complex expression control systems, as summarized in this perspective.

**Viral vectors in gene delivery:** Some of the earliest viral vectors utilized in gene transfer applications were retroviruses, characterized by their ability to integrate stably into the host genome. The prototypical retroviral vector, derived from Moloney murine leukaemia virus, consisted of a ssRNA genome **~**7–11 kb in length, flanked by long terminal repeats that facilitate integration (Ranzani et al., 2013). Upon cell entry, the viral RNA genome undergoes reverse transcription to produce double-stranded DNA, which subsequently integrates into the host chromosome through the action of the viral integrase enzyme. While the genomic integration capability of retroviruses afforded long-term transgene expression, it also posed substantial safety concerns, particularly regarding insertional mutagenesis. The development of self-inactivating vectors with deleted U3 regions in the 3’ long terminal repeat reduced the potential for promoter activation near integration sites, addressing one of the mechanisms that could lead to insertional oncogenesis. Efforts to develop engineered integrases that direct integration to specific genomic sites or identify vector configurations that provide safer integration away from proto-oncogenes were also made. Human immunodeficiency virus type 1-based lentiviruses are a type of retroviruses with enhanced ability to transduce both dividing and non-dividing (e.g. neuronal) cells. Lentiviral vector advantages include their ability to provide long-term transgene expression, sometimes for years in postmitotic neurons, relatively large packaging capacity (8–9 kb), and versatility of cell targeting by virus (tropism) via pseudo-typing with diverse envelope proteins: Vesicular stomatitis virus-glycoprotein offers broad cellular tropism, while rabies virus glycoprotein enables neuronal targeting (Mazarakis et al., 2001). Lentiviral vectors, however, suffered from random integration, and safety issues, like other retrovectors. In addition to integrating lentiviral vectors, integration-deficient lentiviral vectors markedly reduce chromosomal integration and predominantly support transient expression, thereby providing a safer option that avoids genome integration.

Adeno-associated viruses (AAVs) have emerged as the predominant vector system for CNS gene transfer, due to their higher safety profile, efficient neuronal transduction, and minimal immunogenicity (Hudry and Vandenberghe, 2019). AAVs are small non-enveloped parvoviruses with a single-stranded DNA genome of **~**4.7 kb, flanked by inverted terminal repeats that serve as origins of replication and contain packaging signals. The packaging capacity of AAVs ranges from **~**4.4–4.7 kb, depending on the serotype and vector design. AAVs packaged in different serotype capsids (AAV1–9) exhibit different tropism patterns in the CNS due to capsid structural variations affecting cellular entry (binding affinity to receptors, and internalization) and intracellular pathways. For example, AAV2 shows moderate neuronal transduction with limited spread and has advanced to clinical trials for neurodegenerative disorders (Rafii et al., 2014). AAV5 exhibits higher tissue distribution than AAV2 and an ability to transduce both neurons and astrocytes efficiently. AAV8 shows robust neuronal tropism with stable expression. Among the natural capsid serotypes, AAV9 has been the “workhorse” for CNS delivery for several reasons: (i) It possesses the remarkable ability to cross the blood-brain barrier following systemic administration (e.g.intravenous administration), enabling widespread CNS transduction without multiple parenchymal injections, as well as systemic (e.g. PNS, muscle, heart, liver) transduction, which made it suitable for neuro-systemic diseases such as spinal muscular atrophy (Mendell et al., 2017). Blood–brain barrier crossing by AAV9 is strongest in neonates, while adult mice may need higher dosing or cerebrospinal fluid delivery. (ii) AAV9 enables long-lived episomal expression of transgenes in postmitotic neurons, without host genome integration. (iii) AAV9 can be combined with cell-type-specific promoters (e.g., synapsin-1 for neurons, glial fibrillary acidic protein for astrocytes) to regulate the specificity of tissue expression of the transgenes. Although AAV vectors are often described as relatively low in immunogenicity in rodent CNS applications, high-dose systemic administration can elicit high innate and adaptive immune responses. Cerebrospinal fluid delivery, however, showed significantly reduced neutralizing antibody interference and inflammatory responses.

Engineered capsids for AAV vectors have been developed. For example, the AAV-PHP.B vector demonstrated 40-fold higher brain transduction efficiency than AAV9 in C57BL/6J mice. It was later shown, however, that it exhibits strain-specificity, limiting its translation to other mouse strains (Hordeaux et al., 2018). A chimeric capsid serotype (AAV-DJ) generated by DNA shuffling from multiple serotypes exhibits enhanced transduction efficiency across diverse cell types, including liver cells. Limitations of AAV include their limited packaging capacity of ≤ **~**4.7 kb. However, this limitation can be mitigated by tactics such as dual-AAV approaches, trans-splicing of split introns, and/or employing a minimal promoter. Pre-existing neutralizing antibodies to AAV9 are common; however, direct targeting of specific tissues or brain regions where the transgene expression is desired, e.g., via intracerebroventricular or intrathecal injection, overcomes this limitation. Single-stranded AAV preserves the full **~**4.4–4.7 kb packaging capacity but relies on second-strand synthesis by the transduced host cell, which can delay expression onset and reduce peak levels in some conditions. Self-complementary AAV vectors pre-package a double-stranded genome and therefore halve the available cargo capacity (**~**2.3 kb), but they typically mediate faster onset and higher magnitude of transgene expression at a given dose. This trade-off between cargo capacity and expression kinetics is an important design parameter for CNS experiments, particularly when large complementary DNAs or complex expression cassettes are being considered.

Vectors that provide very large packaging capacities include adenovirus (AdV) and herpes simplex virus (HSV) vectors. AdV vectors are derived from non-enveloped double-stranded DNA viruses. First-generation AdV vectors containing viral E1/E3 genomic region deletions allow **~**8.3 kb transgene capacity, and elicit strong immunogenic responses. Second-generation AdV vectors contain additional deletions in E2/E4 regions, reducing inflammatory responses and increasing insert capacity to **~**10.5–12 kb. Third-generation helper-dependent AdV vectors maintain only inverted terminal repeats and packaging signals, offering **~**36 kb capacity with significantly reduced immunogenicity. Still, the clinical application of AdV vectors for CNS disorders remains restricted by immunogenicity concerns (Lowenstein et al., 2007). Classical human Ad5-based vectors are mostly hepatotropic and immunogenic upon systemic administration, whereas canine adenovirus type 2 vectors show strong neuronal tropism and are widely used as robust retrograde tracers in rodent brain when injected into target nuclei.

On the other hand, HSV vectors have a strong affinity for neurons, but also infect progenitor cells and microglia (Deverman et al., 2018). Recombinant attenuated HSV vectors, encompassing large genomic deletions, showed reduced toxicity/immunogenicity while affording up to **~**150 kb transgene inserts. HSV amplicons are plasmid-based vectors containing only the HSV origin of replication and packaging signal and eliminating most viral genes, showing further reduced toxicity and immunogenicity potential; however, they typically provide only transient expression but can be prolonged by incorporating latency-associated elements, yet silencing and heterogeneity between cells and animals remain common challenges. Besides toxicity and immunogenicity concerns, HSV vector usage is complicated by manufacturability and scalability issues (since HSV vector production is a highly tedious and lengthy process), and the uncertainty in the field about the potential long-term consequences of latent virus genomes (Ghosh et al., 2020).

**Delivery routes and administration strategies:** The efficacy of CNS gene delivery depends on vector selection (summarized in **Additional Table 1**) and administration routes which include direct CNS administration and or systemic delivery (summarized in **Additional Table 2**). For example, intracerebroventricular injection delivers vectors directly into cerebral ventricles, utilizing cerebrospinal fluid circulation for CNS-wide distribution. This approach offers widespread distribution of the transgene, technical convenience, and reduced peripheral exposure by concentrating the vector into the CNS. However, intracerebroventricular delivery efficacy varies with animal age, vector serotype, viral dose, and animal species. In neonatal mice, different serotype deliveries were tested to achieve neuronal and glial transduction throughout the CNS (Chakrabarty et al., 2013). Intraparenchymal delivery involves stereotaxic injection directly into brain tissue, providing precise spatial control of transgene expression. The main advantages of this approach are anatomical specificity, efficient vector use, and targeted transduction. However, vector distribution in other regions is typically limited to the injection site(s) and regions connected by axonal transport. The convection-enhanced delivery method can enhance vector distribution through continuous infusion under positive pressure, extending the spread several centimeters from the infusion site. On the other hand, intrathecal injection delivers viral vectors into the subarachnoid space via lumbar puncture or cisterna magna injection. Intrathecal approach has demonstrated improved efficacy for spinal cord transduction in both rodents and non-human primates; however, some toxicity concerns with high viral doses were identified.

**Additional Table 1:** A guide to viral vector choice for gene delivery applications

Characteristic	Retrovirus	Lentivirus	AAV	Adenovirus	HSV
Genome	ssRNA	ssRNA	ssDNA^a^	dsDNA	dsDNA
Packaging capacity	~8 kb	~8-9 kb	~4.7 kb	~8 kb (early generation) - 36 kb (third generation)	~150 kb
Integration	Yes	Yes	No^b^	No	No
Transduces non- dividing cells	No	Yes	Yes	Yes	Yes
Expression duration	Long-term	Long-term	Long-term	Moderate	Moderate to long-term
Immune responses	Low	Low	low at CNS-scale doses; can be high with high-dose systemic delivery	High	Moderate
Retrograde transport	No	Limited natively	Serotype-dependent	Limited	Yes
CNS tropism	Mainly mitotic cells	Neurons, glia	Neurons, glia	Astrocytes, neurons	Neurons, neural progenitors, microglia
Production	Well-establishe d, scalable	Well-establish d, scalable	e Well-established, scalable	Complex but feasible	Very complex
Major limitations	Restricted to dividing cells	Insertional mutagenesis risk	Small cargo size	Residual immunogenicity	Cytotoxicit y, Complex production

^a^ssAAV vs. scAAV (scAAV: faster onset but half cargo size). ^b^While AAV vectors primarily do not integrate, rare integration events were reported at chromosome breakage sites or active genes under certain conditions. AAV: adeno-associated virus; CNS: central nervous system; dsDNA: double-stranded deoxyribonucleic acid; gen.: generation; HSV: herpes simplex virus; scAAV: self-complementary AAV; ssAAV: single-stranded AAV; ssDNA: single-stranded deoxyribonucleic acid; ssRNA: single-stranded ribonucleic acid.

**Additional Table 2:** A guide to delivery route choice for gene delivery applications

Mouse age	Preferred route	IV	CSF (ICV/IT)	Intraparenchymal/CED
Neonatal (P0-P3)	ICV, IV (tail/temporal)	High, widespread CNS	ICV yields broadest CNS spread at low doses	Effective local transduction
Juvenile (P10-P21)	ICV/IT; limited IV	Moderate; declines vs neonatal	Effective CNS	Stable local expression
Adult	IT/ICV; intraparenchymal/CED	Limited for CNS due to BBB	Significant CNS without systemic toxicity	Focal nuclei or defined tracts

BBB: Blood-brain barrier; CED: convection-enhanced delivery; CNS: central nervous system; CSF: cerebrospinal Fluid; ICV: intracerebroventricular; IT: intrathecal; IV: intravenous; P: postnatal day.

For systemic delivery, intravenous injections offer global CNS accessibility and potentially uniform distribution, but require blood-brain barrier crossing by the virus vectors. Engineered vectors (AAV-PHP.B) showed enhanced blood-brain barrier penetrance in some mouse strains, but not necessarily other species (Hordeaux et al., 2018). Intravenous administration of AAV9 has achieved clinical success in treating pediatric monogenic disorders, exemplified by Zolgensma® for type-1 SMA, a severe neurodegenerative disease (Mendell et al., 2017). Systemic delivery, however, is more prone to eliciting proinflammatory or immunogenic responses. For example, unmethylated CpG motifs in the AAV genome activate TLR9, which triggers proinflammatory pathways. However, CpG depletion of vector genomes prevented TLR9 engagement and type I interferon signaling, increasing transgene longevity in preclinical studies (Bertolini et al., 2021). Use of TLR9-inhibitory sequences, CpG-blocking oligodeoxynucleotides, or a corticosteroid course represents additional approaches to curb potential immunogenicity or inflammatory responses. Another aspect to consider for translational relevance is the species differences in immunogenicity. For example, intravenous AAV9 CNS transduction might not necessarily extrapolate to larger species, such as nonhuman primates. Also, engineered capsids such as AAV-PHP.B, which show significant CNS tropism in C57BL/6J mice, rely on Ly6a expression at the blood–brain barrier; Ly6a expression is strain-specific and thus the expression pattern may differ from one strain to another.

In conclusion, for each desired application, both the vector type and the delivery route should be taken into consideration. Moreover, the desired goals and potential risks (e.g., transient *vs.* durable expression, small *vs*. large payload delivery, animal age, systemic exposure, immunogenicity) should all be weighed in. **Additional Tables 1** and **2** summarize a clear roadmap for decision-making for gene delivery to the mouse brain. To aid experimental planning, we summarize these considerations in a decision-tree style schematic (**Figure 1**) that maps cargo size, desired expression duration, target cell type, and extent of CNS expression onto representative vector/route combinations for mouse brain studies.

**Figure 1:**
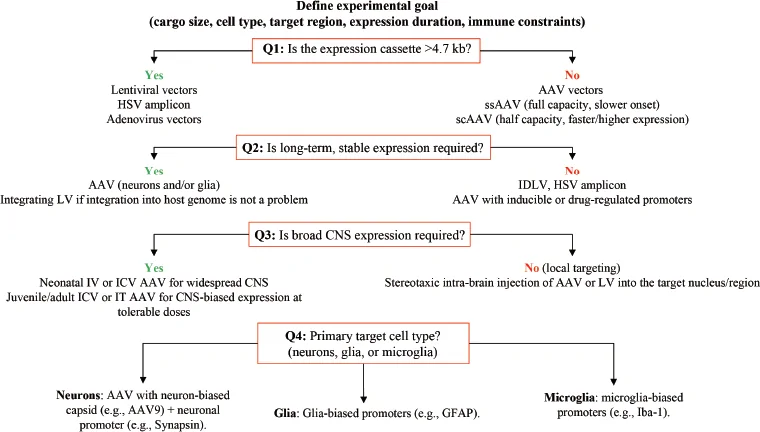
Decision tree for selecting viral vectors for CNS-targeted gene delivery. Decision tree summarizing key considerations for choosing viral vectors for CNS gene delivery in mice. AAV: Adeno-associated virus; AAV9: adeno-associated virus serotype 9; CNS: central nervous system; GFAP: glial fibrillary acidic protein; HSV: herpes simplex virus; Iba-1: ionized calcium-binding adapter molecule 1; ICV: intracerebroventricular; IDLV: integration-deficient lentiviral vector; IT: intrathecal; IV: intravenous; kb: kilobase; LV: lentiviral vector; scAAV: self-complementary AAV; ssAAV: single-stranded AAV.

## Additional files:

***Additional Table 1:*** *A guide to viral vector choice for gene delivery applications.*

***Additional Table 2:*** *A guide to delivery route choice for gene delivery applications.*
